# Livestock animals to study infertility in women

**DOI:** 10.1093/af/vfz017

**Published:** 2019-06-25

**Authors:** Mohamed A Abedal-Majed, Andrea S Cupp

**Affiliations:** 1 Department of Animal Production, School of Agriculture, University of Jordan, Amman, Jordan; 2 Department of Animal Science, University of Nebraska-Lincoln, Lincoln, NE

**Keywords:** follicles, human, infertility, livestock

**Implications**

- Anovulation is a major cause of infertility in mammalian females.
- Ovarian research in humans is challenging due to the shortage of “normal tissue” that can be obtained from women that are trying to get pregnant.
- Livestock animals provide valuable resources to investigate the optimal intra-follicular environment required to facilitate folliculogenesis and translate to humans.
- Different livestock models may be more useful depending on the specific biological process studied.

## Introduction

**Figure 1.**
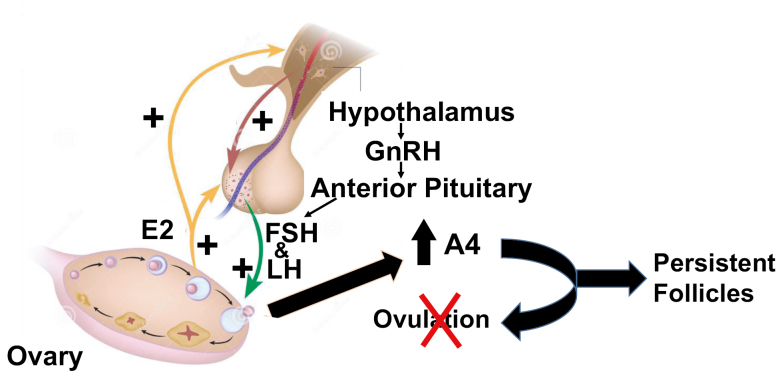
Conceptual model of how androstenedione affects the normal hypothalamic–anterior pituitary–ovarian axis resulting in anovulation. A4, androstenedione; E2, estradiol-17β; FSH, follicle-stimulating hormone; GnRH, gonadotropin-releasing hormone; LH, luteinizing hormone.

Infertility is defined as the inability to achieve pregnancy after 1 yr of regular unprotected intercourse (Smith et al., 2003; Adams et al., 2012). In western countries, one in seven couples will seek medical advice for reproductive failure and infertility affects 8% to 10% of married or cohabiting couples (Allan et al., 1998). A major cause of infertility in mammalian females is anovulation. Endocrine hormone imbalances due to inappropriate regulation of the hypothalamic–pituitary–gonadal axis commonly lead to ovulatory disorders (Figure 1) which account for 25% of couples seeking fertility treatment (Smith et al., 2003). Understanding what affects the ovulation process as well as understanding what might be happening with the complex process of folliculogenesis or atresia would allow for development of innovative strategies to improve female fertility. Moreover, a better understanding of the physiological processes that control ovarian folliclular development and leads to the development of a mature oocyte, within a follicle, would allow for more optimal assisted reproductive technologies in livestock and women (Allan et al., 1998).

## Why Use Livestock as a Model to Understand Anovulation and Female Infertility?

Ovarian research in humans is challenged due to the shortage of normal tissue from women of reproductive age that has not been subjected to large doses of hormones used in vivo for assisted reproductive protocols (Campbell et al., 2003). Animal models that can be manipulated or used to obtain specific cells or tissues are considered valuable as tools to investigate pathophysiological processes of human diseases. Owing to the obvious logistical and ethical limitations on human experimentation, some relevant queries cannot be answered by directly studying affected human patients. In addition, the use of animal models can improve our understanding of the pathophysiology of human diseases and support and test development of innovative therapies or treatments.

For decades, researchers have utilized rodents as a model for human disease and to understand human physiology. As an example of how much rodents are utilized as human models, Adams et al., 2012) reported that 98% of grants from the National Institutes of Health and the Department of Health and Human Services utilized rodents as the model for research. Rodents are a cost-effective model and they have short-generation intervals. Novel technologies can be used to genetically alter or eliminate genes in specific cells of rodents to better understand biological processes critical to biomedical research. However, there are also limitations to the use of rodents as biomedical models. When investigating reproductive processes such as folliculogenesis, the rodent is not as effective a model as other species in translating to human pathologies (Paixao et al., 2017) (Table 1). For example, rodents are considered poly-ovulatory while women are considered mono-ovulatory. Additionally, the onset and development of folliculogenesis is very different between rodents and women (Adams et al., 2012) (Table 1). The rodent reproductive cycle is also very short (i.e., 4 d) when compared with 28 d in women (Table 1). Finally, rodents have a gestation length of 21 d compared with 9 mo in women (Table 1) and it is very difficult to collect sufficient quantities of blood samples from rodents to obtain dynamic patterns of endocrine, metabolic, or steroid hormones for comparison to women (Soncin et al., 2015).

**Table 1.** Reproductive characteristics of humans, ewes, cows, and rodents

Reproductive characteristics	Women	Cows	Ewes	Rodents
Days of gestation that follicle assembly occurs	133	142	100	Shortly after birth
Ovulatory cycle (days)	24–30	17–24	13–19	4–6
Ovulations per cycle	1	1	1–3	12–14
Length of follicular phase (days)	12–14	2–3	2–3	1–3
Length of luteal phase (days)	14–16	15–18	12–14	Depends on whether female engages in copulation
Diameter of ovulatory follicle (mm)	18–20	15–20	5–7	0.9–1.1
Duration of gestation (days)	278–282	278–282	142–148	21

Several different domestic livestock and nonhuman primate models are beneficial to understanding human biological processes. Pigs and humans have anatomical and physiological similarities that make the pig a good model to study human diseases. Pigs are also widely used as biomedical research models since many of their organs are of similar size and function as human organs (Bassols et al., 2014). The digestive system in pigs is similar to humans and obesity/nutrition-related questions can be answered in this livestock model (Pedersen et al., 2013). The pig is a litter-bearing species with a shorter generation interval, so trans-generational questions may be answered in shorter timeframes (Hanson and Skinner, 2016).

The sheep model provides a good resource for addressing underlying mechanisms and developing preventative and treatment options that are likely to be of translational value to humans (Padmanabhan and Veiga-Lopez, 2013) (Table 1). The basic anatomy, physiology, and size of sheep allows for physiological monitoring and sampling devices to be inserted during gestation to monitor vital signs without loss of pregnancy or undue stress. Other species such as cattle are less resilient to this invasive monitoring during gestation and often do not maintain the pregnancy (Padmanabhan and Veiga-Lopez, 2013). Thus, pregnant sheep have been utilized to develop inter-uterine gestational restriction models due to their hardy nature and ability to maintain pregnancies even when they are being used to insert monitoring devices or obtain blood and gestational fluids. Thus, pregnant sheep are a major model used to understand prenatal programming and events leading to parturition (Grigsby, 2016). One major difference in the pig and sheep is that they are poly-ovulatory and women are mono-ovulatory, which presents challenges when trying to understand follicle development, dominance and oocyte development (Table 1).

Monkeys have been used frequently to investigate human diseases since they are nonhuman primates and have 90% to 93% genetic similarity to humans (Bailey, 2014). They have similar biological processes and are excellent models to better understand reproduction, disorders, and disease. Monkeys also have similar gestation length and have an actual menstrual cycle instead of an estrous cycle, and folliculogenesis in monkeys is similar to women. However, collection of tissue in monkeys at specific follicular developmental phases is difficult and monkeys are expensive to house and maintain (Bailey, 2014).

The bovine model system is well suited to understand reproductive disorders in women. Secretion of hormones during the reproductive (estrous) cycle in cattle is very similar to the menstrual cycle in women and both species have multiple follicular waves that result in ovulation of a single ovarian follicle (Adams and Pierson, 1995). The size of ovarian follicle is similar (15 to 20 mm in diameter) between species and similar pathologic conditions can occur in both species (Adams and Pierson, 1995) (Table 1). The period of follicle assembly (in the developing fetus) occurs through day 133 and 142 of gestation in humans and cattle, respectively (Table 1). Furthermore, both species undergo this transition at a similar time point coinciding with mid-gestation (Adams et al., 2012). In humans, the length of the ovarian luteal phase and the length of gestation are also similar to cattle (Table 1). The cow provides an adequate amount of tissue and purified cell populations to conduct experiments, which is often difficult to obtain in women. Cows can be maintained in large groups of genetically similar females under consistent environmental conditions to reduce sources of phenotypic variation. Thus, cattle are valuable models to elucidate the endocrine and local mechanisms controlling both early and terminal stages of ovarian folliclular development in mono-ovulatory species. Experiments conducted in the cow can have direct relevance to ovarian reproductive disorders in women (Campbell et al., 2003).

## Livestock Animals to Study Ovarian Folliclular Development and Ovulatory Dysfunction as a Biomedical Model for Humans

The mechanisms that underlie the regulation of folliculogenesis are still unclear (Juengel et al., 2018) and need extensive study to unravel major regulators that control these processes. Folliculogenesis begins before birth and continues throughout the reproductive lifespan. Ovarian follicles progress from primordial to later stages with only a small percentage developing to preantral and actually ovulating. A major portion of follicles in mammalian species undergoes atresia (Fortune et al., 2000). Since failure to ovulate (anovulation) is the largest problem with reproduction in female mammals, animal models that exhibit disrupted or arrested ovarian follicular development provide valuable resources to investigate the intra-follicular environment that is required to facilitate ovarian folliculogenesis and ovulation in humans (Campbell et al., 2003).

One disorder that displays ovarian follicular arrest and anovulation is polycystic ovary syndrome (PCOS), a multifactorial disorder affecting 9% to 18% (Di Pietro et al., 2015) of reproductive age women. PCOS is often diagnosed when hyperandrogenism and infertility occur due to dysfunctional follicular maturation and anovulation (Figure 1). The PCOS phenotype has been shown to be programmed during fetal development (Dumesic et al., 2007) when exogenous androgens have been administered. However, the etiology of PCOS is still poorly understood. Thus, any information from animal models that have similar characteristics to PCOS may help improve current understanding of the mechanisms of PCOS initiation and development. Women with two of the three criteria are diagnosed with PCOS: 1) ovulatory dysfunction (amenorrhea or oligomenorrhea); 2) hyperandrogenism; and 3) polycystic ovaries. However, several laboratories suggest (Abbott et al., 2002) that androgen excess is the most important criteria in women diagnosed with PCOS and is caused by abnormal theca cell function leading to abnormal ovarian follicular development (Abbott et al., 2002).

A recent review (Paixao et al., 2017) identified about 933 studies using animals as models to study human diseases. They focused on 39 studies that used different animal models to induce PCOS-like symptoms. These 39 studies included 18 studies with androgen-induced stimulation, 9 studies with estrogens or drugs with estrogen action, including endocrine disruptors, and 12 studies with miscellaneous interventions (other than androgen and estrogen treatment to induce PCOS such as transgenic animals or specific strains). In this review, there was a broad difference among the studies concerning hormonal interventions, animal species, and developmental stage at the time of the experiments. However, most of these studies showed ovarian morphological changes, with increases in the number of cystic and antral follicles and the absence or decrease in the number of corpus lutea suggesting follicles were not ovulating and the animals were anovulatory. As mentioned by this review, most of these studies provide important information on physiological processes that are altered and may contribute to anovulation in women. In addition, these studies may help clarify the mechanisms of anovulation in PCOS patients.

## Prenatally Androgenized Animals as a Model for PCOS

A sheep androgenized model has made significant progress in generating new knowledge to increase our understanding of how gestational testosterone administration may result in a PCOS-like phenotype. Female sheep fetuses exposed to testosterone during early to mid-gestation exhibited reproductive defects including ovarian dysfunction and irreversible morphological and functional changes in the reproductive axis after puberty (Forsdike et al., 2007; Steckler et al., 2009). These individuals also had disrupted reproductive cycles and an increase in the proportion of growing follicles in the ovarian cortex during postnatal life along with metabolic characteristics similar to PCOS when compared with nonandrogenized fetuses (Steckler et al., 2009). These studies suggest that reproductive disruptions and PCOS-like symptoms may be caused by exposure to prenatal testosterone excess. The results of this study may be of relevance to understanding ovarian cycle disruption and the etiology in women with PCOS (Padmanabhan and Veiga-Lopez, 2013).

The prenatally androgenized sheep model was used to study ovarian steroidogenesis and follicle formation with different breeds of sheep at day 90 (Comim et al., 2015) and 140 (Smith et al., 2009). Results of these studies suggested that fetal exposure to testosterone may cause advanced timing of ovarian follicle formation. Ovaries from androgenized fetuses collected at 140 d of gestation also observed increased formation of ovarian follicles (Smith et al., 2009). Androgen receptors were present in greater concentrations in primordial and primary follicles in ovaries at day 90 in prenatally androgenized fetuses compared with controls, so it is possible that ovarian follicular formation did not occur until later in fetal development. An interesting shift in steroidogenesis was also detected in the ovaries from day 90 prenatally androgenized fetuses with an increased proportion of ovarian follicles positive for HSD3B2 protein compared with controls. Increased expression of HSD3B2 suggests increased activity of the enzyme and a shift from the delta-5 to the delta-4 pathway of steroid biosynthesis in ovaries from prenatally androgenized fetuses. These data raise key questions about the role of exposure to androgen in utero in developmental origins of PCOS and support the concept that aberrant ovarian follicular development in PCOS is programmed by exposure of the ovary to excess androgen during fetal life.

In addition to changes in ovarian follicular development, prenatal androgenized ovaries had changes in matrix proteins, which have been shown to contribute to development of persistent ovarian follicles. The increase in matrix proteins disrupts the normal tissue remodeling process, availability of different growth factors, and communication through gap junctions. It also causes thickening of the ovarian cortex, which is often seen in women diagnosed with PCOS (Puttabyatappa et al., 2018). Increases in matrix metalloproteinases may be related to increased androgen receptor expression (Ortega et al., 2009). Thus, prenatal androgen exposure may alter ovarian folliculogenesis through direct androgen actions and also actions of androgens on remodeling and structure of the ovarian matrix. These changes in structure of the ovarian matrix may also inhibit the ability of these follicles to mature and to ovulate resulting in the polycystic phenotype.

Exposure to prenatal androgen also results in altered LH secretion, reduced hypothalamic sensitivity to steroid negative feedback, and increased insulin secretion due to increased abdominal adiposity in rhesus monkeys (Dumesic et al., 2007). These monkeys had ovarian morphology similar to PCOS patients with hypergonadotropism and polycystic ovaries. Thus, nonhuman primates exposed to prenatal androgen recapitulate the metabolic and reproductive traits that are often associated with PCOS.

## Naturally Occurring Animal Models of Androgen Excess

Most of the animal models for PCOS are developed via exogenous androgen treatment (Dumesic et al., 2007; Forsdike et al., 2007; Steckler et al., 2009) or increases in diet (Newell-Fugate et al., 2014). There are several animal models that have naturally occurring androgen excess and similar characteristics to women with PCOS. Naturally occurring hyperandrogenism has been described in adult female monkeys with elevated LH and anti-Mullerian hormone, anovulation, poly-follicular ovaries, and infertility (Abbott et al., 2017). Scientists that have identified this population of monkeys do not know whether these hyperandrogenic individuals are present in all monkey populations or the percentage of the population that are affected in most monkey colonies.

An additional naturally occurring androgen excess animal model with PCOS-like characteristics is the High A4 cow model described by Summers et al. (2014). These scientists identified a subpopulation of beef cows that contain excess intrafollicular androstenedione (A4), theca molecular phenotypes (greater mRNA abundance *CYP17A1*, *CYP11A1*, *GATA6*, and *LHCGR*) similar to women diagnosed with PCOS and subfertility, with a 17% reduced calving rate (Summers et al., 2014). Interestingly, these High A4 cows have offspring that are 24 kg heavier at weaning (Summers et al., 2014). Cattle producers often maintain females in the herd that wean heavier calves to increase their profitability by selling more pounds of product. Thus, the High A4 phenotype may have been a result of retaining these females in the herd with heavier weaned calves and selecting for growth over reproduction traits. Investigation of the muscle tissue of these High A4 cows indicates that their myoblasts are not proliferating but are differentiated and may be in cell cycle arrest. The fact that High A4 cows have calves with heavier weaning weights may suggest that the in utero environment the fetus is subjected to inhibits growth of the fetus in utero (through prenatal programming) and there is compensatory growth from birth to weaning, which is similar to the in utero model of women with PCOS (Barnes et al., 2018).

**Figure 2.**
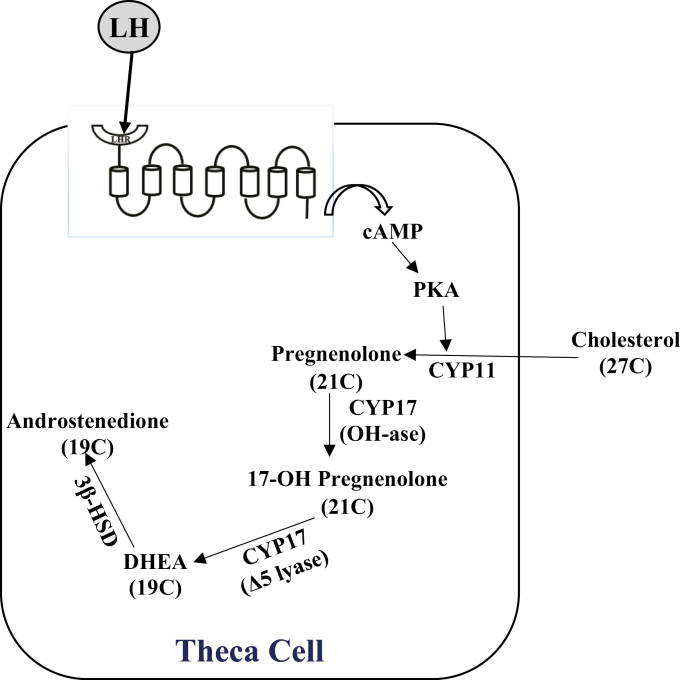
Conceptual model of the normal hormonal steroidogenic pathway in theca cells of the bovine ovary. Cows with excess androstenedione (High A4) have increased expression of several gene (e.g., CYP11A1 and CYP17A1) in the steroidogenic pathway.

In High A4 cows, the excess androgen appeared to be coming from the ovarian theca cells since mRNA abundance for *CYP11A1* and *CYP17A1* (Figure 2) was increased along with mRNA abundance for a transcription factor GATA6, which upregulated promoter regions of both of these steroidogenic enzymes (Summers et al., 2014). In addition to these genes, there was also increased mRNA abundance of LHCGR receptor (Summers et al., 2014) (Figure 2). In theca cell lines derived from women diagnosed with PCOS, microarray data demonstrated increases in *CYP11A1*, *CYP17A1*, *GATA6*, and *LHCGR* receptor (Wood et al., 2004). In addition, the maternal effect gene *ZAR1* was altered in oocytes from High A4 cows when compared with controls in a manner similar to that seen in women with PCOS (Summers et al., 2014) suggesting potential effects on oocyte competence and fertility. Thus, this naturally occurring androgen excess High A4 cow model has a very similar theca and oocyte molecular phenotype to women diagnosed with PCOS and can be used to understand aspects of androgen excess.

**Figure 3.**
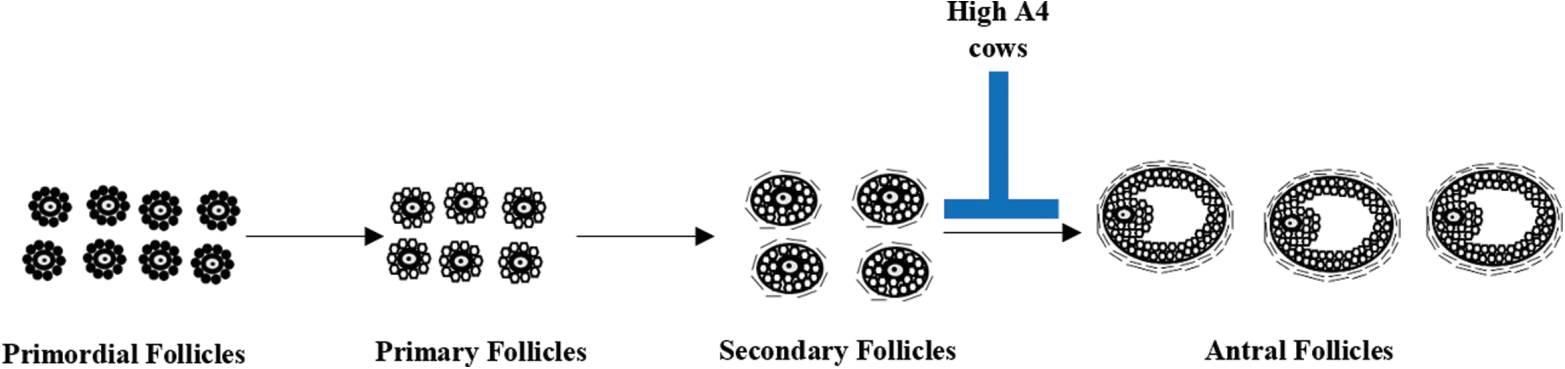
Stages of ovarian follicular development in bovine ovaries. Normal ovarian follicles develop from primordial follicles to primary follicles to secondary follicles to antral follicles, which may ovulate. In cows with excess androstenedione (High A4), secondary ovarian follicles do not develop to the antral follicle stage; thus, ovulation is blocked.

Arrest of ovarian follicles was also a characteristic of this High A4 population of cows where there were reduced numbers of tertiary follicles per section, suggesting arrest of ovarian follicles at the antral stage (Summers et al., 2014) (Figure 3). In addition to increases in A4 in ovarian follicular fluid, there were also increases in testosterone, estrogen, DHEA, 11-deoxycortocosterone, and 11-deoxycortisol, suggesting that steroidogenesis is using the delta-5 pathway normally seen to be upregulated in PCOS patients (Rosencrantz et al., 2011). The High A4 population of cows has been identified in other herds; thus, this model may be more widespread than previously described (Summers et al., 2014). It also appears that the High A4 population cows may constitute 15% to 20% of the herd. Thus, this High A4, natural occurring androgen excess model in beef cattle, could further our understanding of PCOS and provide potential treatment options in the future.

## Conclusions

Domestic livestock have tremendous benefits to provide avenues to conduct biomedical research. These domestic livestock models should be pursued and exploited to provide strategies to increase translation of biomedical research to human diseases. Many diseases and disorders are similar in domestic livestock and humans and with the appropriate approach could be synergistically tested to increase efficiency of application of new knowledge and potential therapies.

## About the Authors

**Dr. Mohamed A. Abedal-Majed** is an assistant professor in Reproductive Physiology and Biotechnology at the Department of Animal Science, University of Jordan, Amman, Jordan. His current research involves studies on the interactions between diet and ovarian follicular development in sheep, estrous synchronization protocols for dairy cows, and determining how ovarian folliculogenesis differs in cows with inherent differences in follicle development and production of androgens. His overall goal is to understand how different environments and nutritional intake affects ovarian folliculogenesis to improve our ability to manipulate ovulation. A better understanding of ovarian folliculogenesis will positively impact animal reproduction worldwide.

**Dr. Andrea Cupp** received her BS in Animal Science from Virginia Tech and her MS and PhD degrees in Reproductive Physiology/Endocrinology from the University of Nebraska–Lincoln (UNL). After a postdoctoral fellowship at the University of California, San Francisco, and Washington State University, she moved to UNL in 2000 as an assistant professor. She is currently the Irvin T and Wanda R Omtvedt Professor of Animal Science. Dr. Cupp’s laboratory uses animal models to understand mechanisms involved in male and female reproductive processes to benefit livestock producers and provide a better understanding of human reproductive disorders and diseases.
